# Linkage of α-cyclodextrin-terminated poly(dimethylsiloxanes) by inclusion of quasi bifunctional ferrocene

**DOI:** 10.3762/bjoc.9.144

**Published:** 2013-07-01

**Authors:** Helmut Ritter, Berit Knudsen, Valerij Durnev

**Affiliations:** 1 Heinrich-Heine-Universität Düsseldorf, Institut für Organische Chemie und Makromolekulare Chemie, Lehrstuhl für Präparative Polymerchemie, Universitätsstraße 1, D-40225 Düsseldorf, Germany

**Keywords:** cyclodextrins, ferrocene, host–guest systems, polysiloxanes, supramolecular chemistry

## Abstract

We report the noncovalent linkage of terminally substituted oligo(dimethylsiloxanes) bearing α-cyclodextrins (α-CD) as host end groups for the cyclopentadienyl rings of ferrocene. This double complexation of unsubstituted ferrocene leads to a supramolecuar formation of the siloxane strands. Structural characterization was performed by the use of ^1^H NMR and IR spectroscopy and by mass spectrometry. Electron microscopy studies and dynamic light scattering measurements show a significant decrease of the derivative size after the complexation with ferrocene. In addition, further evidence for the successful complexation of the end groups was verified by the shifts of the protons in the ^1^H NMR spectra and in the correlation signals of the 2D ROESY NMR spectra.

## Introduction

Polymers containing cyclodextrins (CD) covalently or supramolecularly attached are of increasing interest in recent years. For example, polymers bearing β-CD as side or terminal groups and their interaction with classical guest groups such as adamantane or azo-dyes have been intensively studied [1–4]. The characterization of the inclusion complexes between cyclodextrin and ferrocene as a representative of metallocenes has been the subject of numerous works. Especially Takahashi and Harada were engaged in the analysis of ferrocene complexes with different cyclodextrins [5], which could be obtained in aqueous solution in high yields. In addition, the crystal structure of the α-CD:ferrocene complex has been analyzed by X-ray-diffraction [6]. These studies showed that the ferrocene molecule is encapsulated by two α-CD rings in a tail-to-tail orientation, where all carbon atoms of the cyclopentadienyl rings are in close contact with the cavity of the cyclodextrin macrocycles. The size of the ferrocene molecule is too large to penetrate completely into the α-CD cavity. Only a single cyclopentadienyl ring of the ferrocene is included. The binding mode, thermal stability, formation constants, and dielectric properties [7] of this inclusion complex were also investigated by circular dichroism spectroscopy [8] and thermogravimetric analysis [9]. In addition, the complex of ferrocene and α-CD could be detected in the gas phase by mass spectrometry [10]. Recently, there have been many reports focusing on the use of α-CD in aqueous solution as a mobile phase for optical resolution of ferrocene derivates [11–12]. α-CD-functionalized siloxanes, however, have rarely been described in the literature. There are many reports on siloxanes bearing β-CD attached on the surface, which are used as stationary phases for chromatography and for drug-release systems [13–22]. In a previous paper we described the noncovalent AA–BB-type linkage of poly(dimethylsiloxanes) containing terminal ß-CD groups with terminally attached guest molecules on the poly(dimethylsiloxanes) [23]. With this in mind, the aim of our present study was to synthesize α-CD-terminated poly(dimethylsiloxanes) to create novel supramolecular linear or macrocyclic siloxan structures based on double inclusion complexes with ferrocene.

## Results and Discussion

**Scheme 1:**
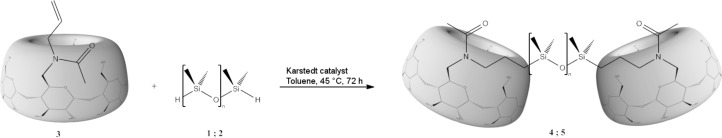
Hydrosilylation of Si–H terminated poly(dimethylsiloxanes) **1** and **2** with mono-((6-*N*-(allylamino)-6-deoxy)-peracetylated-α-CD (**3**) to compounds **4** (*n* = 1) and **5** (*n* = 6–37).

The syntheses of α-CD-terminated poly(dimethylsiloxanes) **4** and **5** were performed by hydrosilylation of mono-((6-*N*-allylamino)-6-deoxy)-peracetylated-α-CD (**3**) using a short-chain oligo(dimethylsiloxane) **1** or a long-chain poly(dimethylsiloxane) **2**, respectively, (Scheme 1).

**Figure 1:**
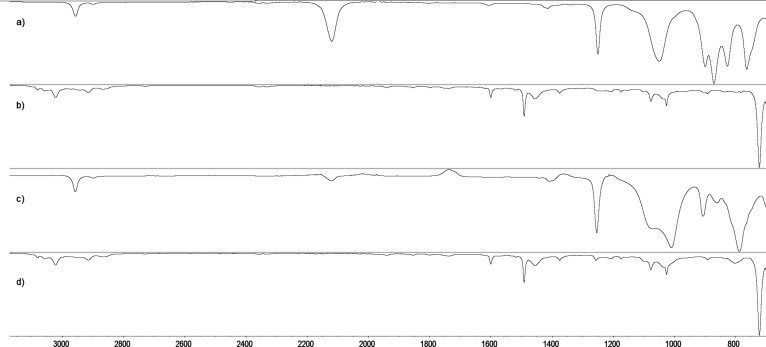
IR spectra of (a) H-terminated disiloxane (**1**), (b) α-CD-terminated disiloxane (α-CD-disiloxane) (**4**), (c) H-terminated polydimethylsiloxane (**2**) and (d) α-CD-terminated polydimethylsiloxane (α-CD-PDMS) (**5**).

During the reactions, samples were collected and investigated by IR spectroscopy in order to ensure the full conversion of the reactants to products (Figure 1).

The disappearance of the absorption bands at 2100 cm^−1^ clearly shows the successful hydrosilylation of the educts to the newly synthesized derivatives **4** and **5**.

The ^1^H NMR spectra of the α-CD-functionalized poly(dimethylsiloxanes) (α-CD-DS **4** and α-CD-PDMS **5**) reveal the characteristic signals of the Si–CH_3_ groups of the siloxane backbones at 0 ppm. In addition, the signals of the cyclodextrin-substituents appear in the area of 3.50 to 6.00 ppm. New peaks at 0.43 and 1.25 ppm of the resulting dimethylene bridge between the siloxane and α-CD are detectable. Signals for the double bond of the vinyl group and the Si–H bond of the starting materials are absent, as expected. The MALDI–TOF spectra of the modified poly(dimethylsiloxanes) **4** and **5** show terminally substituted products of various chain lengths, wherein the distance of 74 g/mol between the mass signals exactly corresponds to one dimethylsiloxane unit. The results of the IR, ^1^H NMR and mass spectroscopy strongly support the existence of the described derivatives. The terminally α-CD-functionalized poly(dimethylsiloxanes) **4** and **5** were subsequently used as host molecules component for the following complexation reactions.

### Host–guest interactions of the siloxane derivatives with ferrocene

Linking of the obtained polydimethyl and disiloxane derivatives through host–guest interactions with single ferrocene molecules generates supramolecular, connected siloxane chains. The guest molecule ferrocene was therefore dissolved in chloroform and treated with the α-CD-siloxane derivatives. The solutions were stirred overnight and analyzed by ^1^H NMR and 2D ROESY NMR.

**Figure 2:**
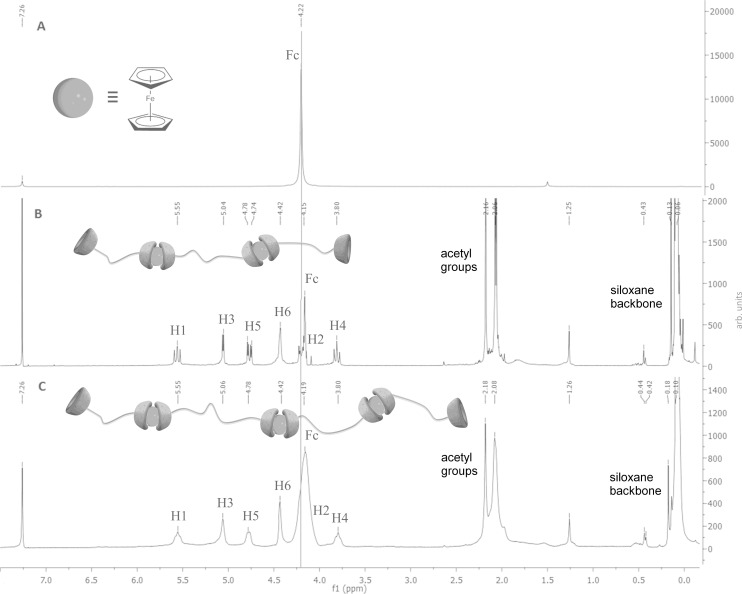
^1^H NMR spectra of ferrocene (A), complex of α-CD-disiloxane (α-CD-DS) **4** with ferrocene (B) and complex of α-CD-poly(dimethylsiloxane) (α-CD-PDMS) **5** with ferrocene (C).

As seen in Figure 2, ^1^H NMR spectra of pure ferrocene and the resulting complexes are compared with regard to the proton signals of the cyclopentadienyl rings of the ferrocene. In the case of pure ferrocene (A) the signal of these protons appears at 4.22 ppm. After the complexation with the α-CD-modified disiloxane **4**, a shift of the cyclopentadienyl signals to 4.15 ppm can be observed (B). This phenomenon is explained by the penetration of the attached cyclopentadienyl rings of the guest molecule ferrocene into the cavity of the cyclodextrin and a subsequent change of the chemical environment of the cyclopentadienyl rings. The same studies were performed with the complex of α-CD modified polydimethylsiloxane **5** and ferrocene (C) to prove a successful complexation. As expected, a clear shift of the ferrocene signal from 4.22 to 4.19 ppm can be detected, which also suggests that ferrocene molecules are completely complexed by the α-CD units.

The question about the formation of linear or macrocyclic suprastructures, cannot be answered by this method. Thus, the various polysiloxane strands can be linked with each other through the host–guest interactions of the terminal α-CD units and pure ferrocene without the need for covalent bonds. In order to complete the characterization of these host–guest interactions of the novel compounds, they were also examined by 2D ROESY NMR spectroscopy.

**Figure 3:**
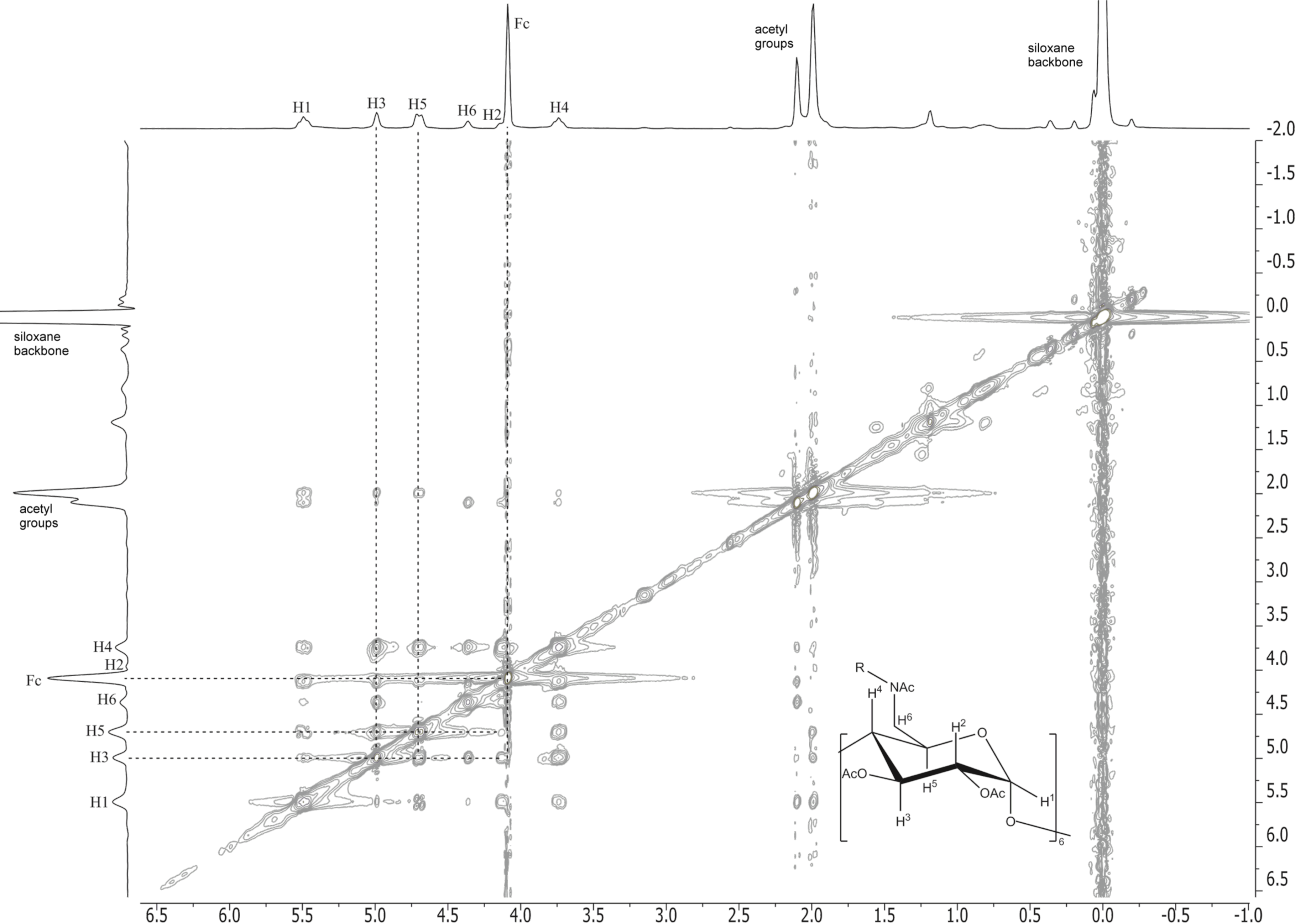
2D ROESY NMR spectra of the complex of α-CD-disiloxane (α-CD-DS) **4** with ferrocene.

**Figure 4:**
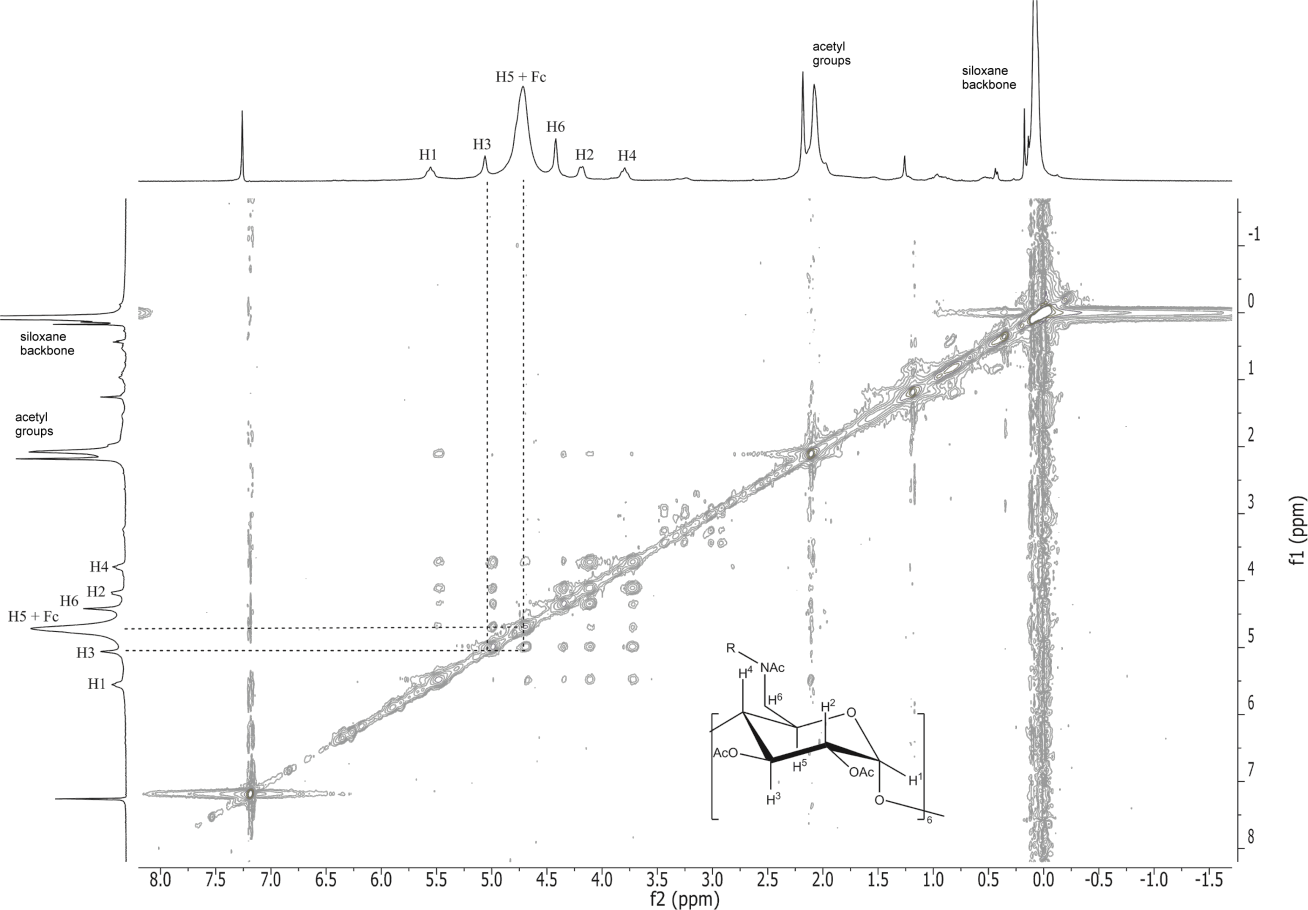
2D ROESY NMR spectra of the complex of α-CD-polydimethylsiloxane (α-CD-PDMS) **5** with ferrocene.

The 2D ROESY NMR spectra of the short-chain α-CD-disiloxane **4** with ferrocene (Figure 3) shows the correlation between the inner cavity protons H-3 and H-5 of the attached α-cyclodextrins and the protons of the cyclopentadienyl rings of the guest molecule ferrocene (Fc) (dotted lines) based on the characteristic cross-peaks of these protons (spots). The 2D ROESY NMR spectrum of the long-chain α-CD-polydimethylsiloxane **5** with ferrocene (Figure 4) also indicates the described proton interactions of the host and guest molecules. The noncovalent bonds of the siloxanes with the ferrocene leading to supramolecular structures are hereby established.

Thin-layer chromatography studies (toluene/ethanol 30:1 by volume) also indicate the presence of enclosed ferrocene in the cavity of the cyclodextrins. UV-active areas can be detected, which do not migrate with the solvent front in contrast to pure ferrocene.

Certainly, the viscosity of the poly(dimethylsiloxane) educts and the complexed systems is of great interest. However, a significant increase of the viscosity in solution could not be detected under the applied conditions, probably due to the relatively high shear forces, which cause the decomplexation of the derivatives mechanically.

**Figure 5:**
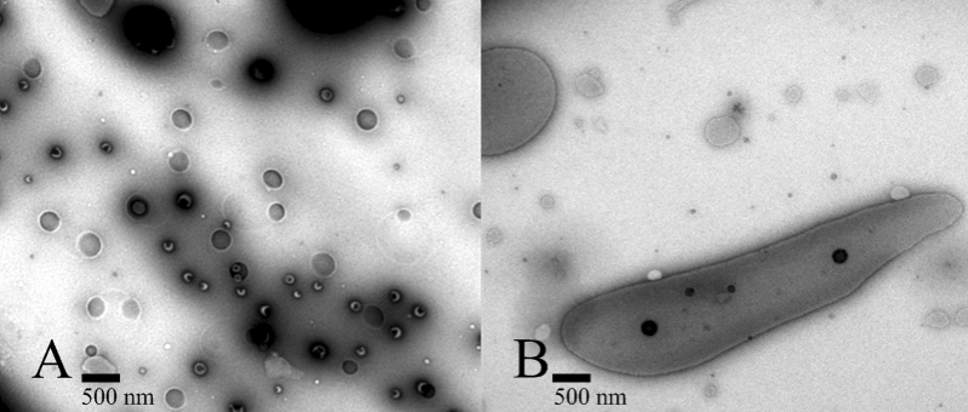
TEM images of α-CD-disiloxane **4** (A) and the supramolecular formation of α-CD-disiloxane **4** with ferrocene (B).

In addition, the successful complexation of the α-CD-modified siloxanes and the formation of supramolecular structures via inclusion complexes with ferrocene can be illustrated in the TEM images of **4** (Figure 5). Figure 5A (α-CD-disiloxane **4**) shows a majority of globular structures of up to 400 nm. In the case of the complex of the compound **4** with ferrocene (Figure 5B), elongated structures of up to 4000 nm can be observed. This phenomenon might be explained by the supramolecular linkage of the short-chain disiloxanes by interaction with the bifunctional ferrocene molecules, which leads to the formation of the larger structures.

**Figure 6:**
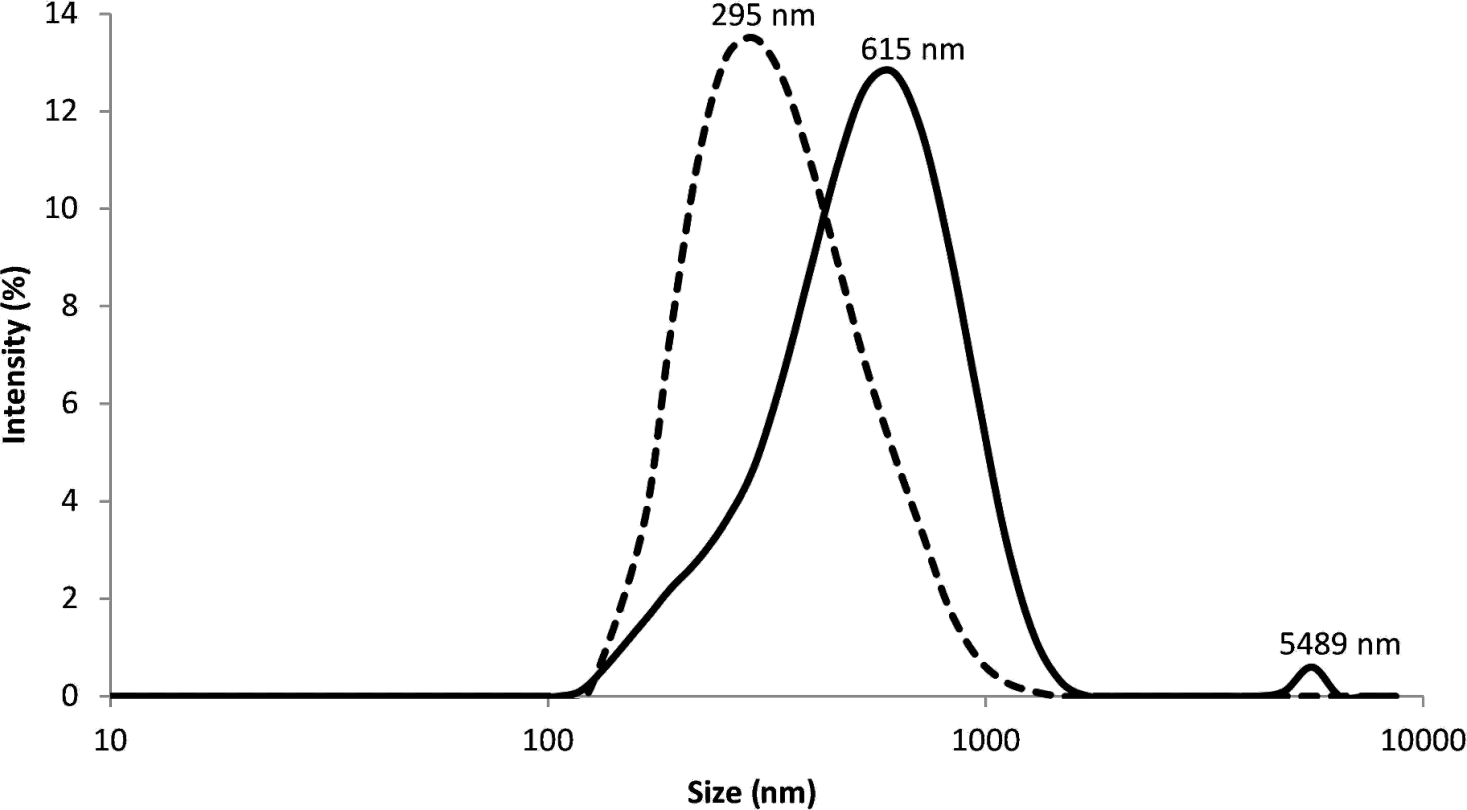
DLS measurements of α-CD-disiloxane **4** (A) (dashed line) and the supramolecular formation of α-CD-disiloxane **4** with ferrocene (B) (solid line).

Finally, DLS (dynamic light scattering) analysis of **4** and **5** and their complexes with ferrocene were performed. Figure 6 illustrates the hydrodynamic diameters of α-CD-disiloxane **4** and α-CD-disiloxane with ferrocene. The average hydrodynamic diameter of α-CD-modified disiloxane **4** (dashed line) can be determined as 343 nm, which corresponds to the average sizes of the particles observed by TEM measurements. After the addition of ferrocene, the resulting complex with **4** was investigated by DLS (solid line) as well. The hydrodynamic diameter significantly shifts from 343 nm to 615 and 5489 nm. The hydrodynamic diameter of 615 nm can be explained by the formation of some macrocycles and oligomers by noncovalent interactions of the host and guest molecules. This phenomenon might also explain the almost unchanged viscosity increase of the complex in comparison to the uncomplexed educts, which is not influenced decisively by the presence of the smaller supramolecular structures. However, the peak at about 5000 nm indicates the presence of larger linear structures based on the complexation of α-CD-disiloxane **4** with the ferrocene molecules, which were also observed in the TEM images.

This further confirms the assumption that the terminally functionalized poly(dimethylsiloxanes) can assemble to supramolecular structures through noncovalent interactions.

## Conclusion

We have demonstrated, for the first time, a chain extension of poly(dimethylsiloxanes) in chloroform as solvent, by host–guest interactions of terminally attached α-CD with ferrocene as a quasi-bifunctional single guest molecule. The formation of noncovalent linkages was proved by the use of NMR spectroscopy and TEM images. However, it turned out that the stability of linking functions is relatively poor with respect to shear forces during viscosity measurements.

## Experimental

### Materials and methods

Commercial reagents and solvents were purchased from ABCR and Sigma Aldrich and used as received. When necessary, solvents were dried and purified by appropriate standard procedures. Mono-(6-(*p*-toluenesulfonyl)-6-deoxy)-α-cyclodextrin (α-tosyl-CD), mono-(6-*N*-allylamino)-6-deoxy)-α-cyclodextrin (AAm-α-CD) and mono-((6-*N*-allylamino)-6-deoxy)-peracetylated-α-cyclodextrin (AAm-Ac-α-CD) [21] were synthesized according to the literature. MALDI–TOF mass spectra were recorded with a Bruker Ultraflex time-of-flight mass spectrometer. The device operates both in the linear mode and in reflector mode with a 337 nm nitrogen laser. The samples were dissolved in a suitable solvent. A dithranol (DIT) or 2,5-dihydroxybenzoic acid (DHB) matrix was used. The IR spectra were recorded with a Fourier transform infrared spectrometer FTIR Nicolet 5SXB. The calibration of the wave numbers was done by using a He–Ne laser. The ATR measurements were performed with a Specac Golden Gate diamond-ATR unit. For the NMR measurements at 300 MHz a Bruker Avance III - 300 (2010) spectrometer was used. The magnetic field strength was 7.05 T. This corresponds to an absorption frequency of 300 MHz for ^1^H NMR and 75 MHz for ^13^C {^1^H} NMR. For ESI-measurements on an ion-trap mass spectrometer API Finnigan LCQ Deca, the substances were dissolved in a suitable solvent at a concentration of 2 mg·mL^−1^. The ionization was carried out by electrospray ionization (ESI). The TEM images were obtained on a Zeiss EM 902A microscope at 80 kV. The samples were dissolved in chloroform and dripped on copper grids (Formvar/carbon film 400 mesh). After evaporation of the solvent, the remaining pure substances could be examined under a high vacuum (10^−7^ bar). The hydrodynamic diameters of the derivatives and their complexes were determined by use of dynamic light scattering (DLS) in the back scattering mode with a Malvern Zetasizer Nano ZS ZEN3600 at a laser wavelength of 633 nm and a detection angle of 173°. The samples were dissolved in chloroform (10 mg·mL^−1^) and measured in quartz cuvettes of 1 cm thickness. The measurement data were evaluated with a nonnegative least squares (NNLS) algorithm. The number-averaged diameters were used to characterize the samples.

### Synthesis of siloxane-derivatives

**Synthesis of mono-((6-*N*-allylamino)-6-deoxy)-peracetylated-α-cyclodextrin-dimethyldisiloxane (α-CD-disiloxane) (4):** Mono-((6-*N*-allylamino)-6-deoxy)-peracetylated-α-cyclodextrin (0.70 g, 0.40 mmol) is dissolved in 30 mL of dry toluene under an argon atmosphere at 45 °C. 1,1,3,3-Tetramethyldisiloxane (0.04 mL, 0.20 mmol) is added. In a period of 5 h, the Karstedt catalyst (50 µL, 2.1–2.4% Pt) is added in portions. After 72 h, the toluene is evaporated and the product is obtained by fractional precipitation as a solid (0.20 g, 20%). IR: 3026, 2919, 1746, 1605, 1495, 1459, 1260, 1140, 1081, 1029 cm^−1^; ^1^H NMR (300 MHz, CDCl_3_) δ 0.00 (m, 12H), 0.43 (m, 4H), 1.25 (s, 4H), 2.06–2.16 (m, 108H, CH_3_CO), 3.80 (m, 12H), 4.15 (m, 12H), 4.42 (m, 24H), 4.74–4.78 (m, 12H), 5.04 (m, 12H), 5.55 (m, 12H); ESI–MS *m*/*z*: 3148.34 [M + Na]^+^.

**Synthesis of mono-((6-*N*-allylamino)-6-deoxy)-peracetylated-α-cyclodextrin-polymethylsiloxane (α-CD-PDMS) (5):** Mono-((6-*N*-allylamino)-6-deoxy)-peracetylated-α-cyclodextrin (0.70 g, 0.4 mmol) is dissolved in 60 mL of dry toluene under an argon atmosphere. Hydride-terminated poly(dimethylsiloxane) (0.3 mL, 0.22 mmol) is added and heated to 75 °C. The Karstedt catalyst (100 µL, 2.1–2.4% Pt) is added in portions over 1 h. After 72 h, the solvent is evaporated and the product is obtained by fractional precipitation as a solid (0.40 g, 35%). IR: 3026, 2919, 1746, 1605, 1495, 1459, 1260, 1140, 1081, 1029 cm^−1^. ^1^H NMR (300 MHz, CDCl_3_) δ 0.00 (m, 78H), 0.44–0.42 (m, 4H), 1.26 (s, 4H), 2.06–2.16 (m, 108H, CH_3_CO), 3.80 (m, 12H), 4.19 (m, 12H), 4.42 (m, 24H), 4.78 (m, 12H), 5.06 (m, 12H), 5.55 (m, 12H); MALDI–TOF–MS *m*/*z*: 3962.58 [M + Na]^+^ for *n* = 12.

### Complexation reactions

**Complexation of α-CD-disiloxane (4) with ferrocene:** α-CD-disiloxane (100 mg, 0.015 mmol) is dissolved in a solution of ferrocene (2.8 mg, 0.015 mmol) and 2.0 mL of chloroform and stirred at rt overnight. To isolate the product, the solvent is evaporated. The complex is obtained as a solid. ^1^H NMR (300 MHz, CDCl_3_) δ 0.01–0.16 (m, 12H), 0.44–0.42 (m, 4H), 1.25 (s, 4H), 2.06–2.16 (m, 108H, CH_3_CO), 3.69–3.85 (m, 2H), 4.15 (s, 10H, ferrocene), 4.25 (m, 2H), 4.42 (m, 2H), 4.74–4.78 (m, 38H), 5.04 (m, 14H), 5.55 (m, 14H).

**Complexation of α-CD-PDMS (5) with ferrocene:** α-CD-PDMS (40 mg, 0.01 mmol) is dissolved in a solution of ferrocene (1.5 mg, 0.01 mmol) and 2.0 mL of chloroform and stirred at rt overnight. To isolate the product, the solvent is evaporated. The complex is obtained as a solid. ^1^H NMR (300 MHz, CDCl_3_) δ 0.01–0.16 (m, 12H), 0.44–0.42 (m, 4H), 1.25 (s, 4H), 2.06–2.16 (m, 108H, CH_3_CO), 3.69–3.85 (m, 2H), 4.19 (s, 10H, ferrocene), 4.25 (m, 2H), 4.42 (m, 2H), 4.74–4.78 (m, 38H), 5.04 (m, 14H), 5.55 (m, 14H).
